# Molecular subtyping of DCIS: heterogeneity of breast cancer reflected in pre-invasive disease

**DOI:** 10.1038/sj.bjc.6606021

**Published:** 2010-12-07

**Authors:** S E Clark, J Warwick, R Carpenter, R L Bowen, S W Duffy, J L Jones

**Affiliations:** 1 Centre for Tumour Biology, Institute of Cancer and CR-UK Clinical Centre, Barts and the London School of Medicine and Dentistry, John Vane Science Centre, Charterhouse Square, London EC1M 6BQ, UK; 2 Cancer Research UK Centre for Epidemiology, Mathematics, and Statistics, Wolfson Institute of Preventive Medicine, Barts & The London School of Medicine and Dentistry, Queen Mary University of London, Charterhouse Square, London EC1M 6BQ, UK; 3 Breast Unit, St Bartholomew's Hospital, West Smithfield, London EC1A 7BE, UK

**Keywords:** DCIS, intrinsic subtypes, basal, triple negative, Bcl-2.

## Abstract

**Background:**

Molecular profiling has identified at least four subtypes of invasive breast carcinoma, which exhibit distinct clinical behaviour. There is good evidence now that DCIS represents the non-obligate precursor to invasive breast cancer and therefore it should be possible to identify similar molecular subtypes at this stage. In addition to a limited five-marker system to identify molecular subtypes in invasive breast cancer, it is evident that other biological molecules may identify distinct tumour subsets, though this has not been formally evaluated in DCIS.

**Methods:**

Tissue microarrays were constructed for 188 cases of DCIS. Immunohistochemistry was performed to examine the expression patterns of oestrogen receptor (ER), progesterone receptor (PR), Her2, EGFR, cytokeratin (CK) 5/6, CK14, CK17, CK18, *β*4-integrin, *β*6-integrin, p53, SMA, maspin, Bcl-2, topoisomerase II*α* and P-cadherin. Hierarchical clustering analysis was undertaken to identify any natural groupings, and the findings were validated in an independent sample series.

**Results:**

Each of the intrinsic molecular subtypes described for invasive breast cancer can be identified in DCIS, though there are differences in the relative frequency of subgroups, in particular, the triple negative and basal-like phenotype is very uncommon in DCIS. Hierarchical cluster analysis identified three main subtypes of DCIS determined largely by ER, PR, Her2 and Bcl-2, and this classification is related to conventional prognostic indicators. These subtypes were confirmed in an analysis on independent series of DCIS cases.

**Conclusion:**

This study indicates that DCIS may be classified in a similar manner to invasive breast cancer, and determining the relative frequency of different subtypes in DCIS and invasive disease may shed light on factors determining disease progression. It also demonstrates a role for Bcl-2 in classifying DCIS, which has recently been identified in invasive breast cancer.

DCIS is the non-obligate precursor of invasive breast cancer and accounts for an increasing proportion of breast cancer, particularly in the screening setting. In the UK NHS Breast Screening Programme for 2008–2009, 14 166 new breast cancers were detected, of which 20% were DCIS (NHSBP 2008–2009; http://www.ic.nhs.uk/webfiles/publications/breast_screening/ Breast_Screening_Programme_Report_2008_09.pdf). Despite the increased frequency of detection, the biology of DCIS is poorly understood. A number of histopathological classifications have been proposed to distinguish between different types of DCIS (reviewed in the study by Jones, 2006). The classification system most widely used is based on nuclear morphology, yielding three categories of high-, intermediate- and low-nuclear grades. This classification has some prognostic power with greater risk of recurrence being associated with high-nuclear grade; however, poor reproducibility limits the clinical value of this system.

In invasive breast cancer, recent advances have led to an emerging molecular classification based more on the biological characteristics of the tumour rather than limited to morphological analysis. The seminal work of Perou *et al* (2000) identified novel subtypes of invasive breast cancer based on an intrinsic gene signature established by cDNA microarray analysis. These subgroups have been refined by Sorlie *et al* (2001) and shown to have prognostic significance. Many studies have aimed to identify an immunohistochemical profile that can act as a surrogate for gene array analysis (Makretsov *et al*, 2004; Nielsen *et al*, 2004; Matos *et al*, 2005), and it appears that a five-marker panel of oestrogen receptor (ER), progesterone receptor (PR), Her2, CK5/6 and EGFR shows promise in its ability to categorise invasive cancers to their molecular subtype (Cheang *et al*, 2008).

Much less attention has focussed on dissecting the biological subtypes of DCIS, and there are discrepancies in the results of those studies that exist. Thus, whereas several studies report the existence of a basal subtype of DCIS (Livasy *et al*, 2007; Paredes *et al*, 2007; Tamimi *et al*, 2008), one gene array study found no good evidence of this category of DCIS (Hannemann *et al*, 2006). There are also discrepancies in the relative frequency of subtypes between the *in situ* and invasive disease.

Whereas it has been recognised for some time that there is a higher frequency of Her2-positive DCIS compared with Her2-positive invasive breast cancer (Park *et al*, 2006), there are several reports suggesting that, if a basal category of DCIS exists, it is less frequent than basal type invasive carcinoma, though others suggest a very similar frequency (Livasy *et al*, 2007; Meijnen *et al*, 2008; Tamimi *et al*, 2008). It is important to establish these relationships as they might help to explain the biological factors determining progression of DCIS to invasive disease.

Although a five-marker panel has been identified to categorise breast cancer (Cheang *et al*, 2008), the biological determinants underlying the different clinical behaviour between the molecular subtypes is clearly more complex than implied by such a restricted biomarker panel. In support of this, a number of studies have identified further biologically relevant markers that associate either with a particular subtype or reveal heterogeneity within a subtype. Thus, P-cadherin has been shown to significantly associate with the basal subtype, whereas *β*4-integrin is expressed by a subset of basal tumours (Paredes *et al*, 2007; Lu *et al*, 2008). Certain markers have been shown to have prognostic or predictive importance in invasive breast cancer (Umekita *et al*, 2002; Fritz *et al*, 2005; Malamou-Mitsi *et al*, 2006; Rolland *et al*, 2007), but only infrequently have these been applied to DCIS (Shpitz *et al*, 2000), and a more extensive analysis of the relationship between these biomarkers and their ability to define different subgroups of DCIS of potential clinical relevance is lacking.

This study therefore aimed to analyse the expression profile of 16 biomarkers, shown to be biologically relevant in invasive breast cancer, in a series of 188 cases of pure DCIS. Hierarchical clustering analysis was performed with the aim of identifying molecular subtypes of DCIS and how these relate to recognised subtypes of invasive breast cancer. The analysis was validated on an independent series of DCIS samples. Follow-up data were collected for each case to investigate the relationship between DCIS expression profile and outcome.

## Patients and methods

### Patient selection and tissue samples

For the test series, all patients treated for DCIS without associated invasive carcinoma at St Bartholomew's Hospital, London, UK between 1994 and 2007 were identified. Patients with DCIS associated with LCIS and Paget's disease of the nipple were included in this study. Tumours involving areas of microinvasion were excluded. For the validation series, a further 75 cases of DCIS were identified, diagnosed since 2007. Formalin-fixed paraffin-embedded tissue blocks were retrieved for each patient and used to construct a tissue microarray (TMA; Beecher MTA1 machine (Beecher Instruments Inc., Silver Springs, MD, USA); Alphelys TMA designer, Alphelys, Plaisir, France). Appropriate areas of DCIS were identified by a consultant histopathologist (JLJ) and three × 1 mm cores of DCIS were taken from each case. Cores of normal breast tissue were also included in the array as controls. From the arrays, 4 *μ*m sections were cut and mounted on negatively charged glass slides. Follow-up data and information on treatment, age at diagnosis, histological grade and extent of disease were collected for all patients using the medical and nursing notes and electronic patient record system. Estrogen receptor testing was not routinely performed on all cases from earlier years in the study, but for those that had undergone testing the ER status reported on a full section by the St Bartholomew's histopathologist was recorded in order to compare with the scores from the TMAs. The study was granted NHS Research Ethics Committee approval (COREC No. 06/Q0403/182).

### Immunohistochemistry

Sections were dewaxed in xylene for 5 min and rehydrated through graded alcohols to distilled water. Optimal antigen retrieval procedure and dilutions applied for each antibody are summarised in Supplementary Table 1. Antibodies were used to detect ER (Novocastra, Milton Keynes, UK, NCL-L-ER6F11), PR (Novocastra, NCL-L-PGR 1A6), Her2 (Novocastra, NCL-CBe-356 clone 10A7), EGFR (Dako, Cambridgeshire, UK, K1492), CK 5/6 (Dako, D5/16B4), CK14 (Serotec, Kidlington, UK, LL002), CK17 (Sigma, Sigma-Aldrich, Dorset, UK, CK-E3), CK18 (Serotec, CY90), SMA (Dako, 1A4), p53 (Novocastra, NCL-p53-DO7), topoisomerase II*α* (Novocastra, 3F6), Bcl-2 (Abcam, Cambridge, UK, 100/D5), maspin (Pharmingen, Oxford, UK, G167-70), *β*4-integrin (Chemicon, Watford, UK, W439-9B), *β*6-integrin (Gift from J Marshall 62G2) and P-cadherin (BD Transduction Labs, 56, Oxford, UK). The primary antibodies were diluted in a blocking solution containing the appropriate animal serum and 0.1% bovine serum albumin. Slides were incubated in the primary antibody for 2 h at room temperature or overnight at 4°C. After washing the slides, the appropriate secondary antibody was applied and slides were incubated for 30 min at room temperature. Further, they were washed, and then Streptavidin ABC complex (Dako K0377) was prepared according to manufacturer's instructions and added to the slides for 30 min. Antibody was detected with diaminobenzidine tetrahydrochloride substrate buffer (Vector SK-4100, Vector Labs Inc., Burlingame, CA, USA) prepared according to manufacturers’ instructions. After 5 min incubation, sections were washed, counterstained with haematoxylin, dehydrated and mounted. Each run included appropriate positive and negative controls.

### Interpretation of staining

The staining patterns were scored according to the percentage of DCIS tumour cells staining positive for each antibody. The same scoring system was used for all markers to allow direct comparison between the markers. Samples were either scored as positive when >70% of cells expressed the marker, heterogeneous when 10–70% of cells expressed the marker, occasional when a few individual cells expressed the marker or negative when no staining was seen. For analysis, staining of >10% was considered as positive. Only appropriately located staining for a given marker was scored, that is, nuclear for ER and PR, membrane staining for Her2.

Two independent researchers performed the scoring (SEC and JLJ). All samples were coded before scoring, therefore researchers were blinded to outcome. Comparison was made between the two observers results and, when in conflict, the core was reviewed together and a consensus score agreed.

### Statistical analysis

#### Tetrachoric paired correlation

Tetrachoric correlation was used to measure the degree of association between pairs of markers. An absolute value for the correlation coefficient between 0.1 and 0.3 was taken to indicate weak correlation, between 0.3 and 0.5 to indicate moderate correlation and between 0.5 and 1 to indicate strong correlation.

#### Cluster analysis

Natural groupings in the data were identified using unsupervised hierarchical agglomerative cluster analysis with average linkage on the 16 potential biomarkers. Biomarkers were dichotomised (1=positive, 0=negative) to create binary variables with positive and heterogeneous staining being coded as positive and occasional or negative being coded as negative. Simple matching was used to measure the distances between the clusters. The optimal number of clusters for any given set of biomarkers was determined with reference to both the Calinski, (1973) and Duda (1973) stopping rules.

#### Fisher's exact test

The Fisher's exact statistic (used instead of a *χ*^2^ when the expected value in any cell was <5) was used to test for an association between group (as determined by the cluster analysis) and histological grade.

## Results

### Patients and tumour characteristics

In the test series, a total of 248 women were diagnosed with pure DCIS at St Bartholomew's Hospital between 1994 and 2007. These were all newly diagnosed either through the screening programme or as symptomatic cases. Tissue samples were available for 188 of these and were used to construct the tissue microarrays. Those samples not available were either missing from the file, or the tissue paraffin blocks had been damaged during storage. In two cases, the patient had only undergone a core biopsy but no definitive treatment. The tumour characteristics and treatment of these 188 women are shown in Table 1. The average age at diagnosis was 56 (range 25–85) years. Breast conservation was the most frequently used primary surgical treatment (59.6%) and was combined with a sentinel node procedure in only 10% of these cases. Mastectomy (with or without sentinel lymph node biopsy or axillary clearance) was performed in 34% of cases.

The average lesion size was 25 mm (range 4–155 mm), and 73.9% of the tumours were high grade (HG), 11.2% intermediate grade (IG) and 10.6% low grade (LG). Tumour grade was not known in 1.6% of cases. Where the tumour was of mixed histological grade, the highest was used to define the case. Three of the DCIS cases had occurred in a radial scar and one in a fibroadenoma, three were associated with Paget's disease of the nipple and two contained areas of LCIS. Adjuvant treatment was mostly endocrine therapy (42%). In all, 22 women underwent radiotherapy either in combination with endocrine therapy or alone. Details of adjuvant treatment were not available for 20 women.

Follow-up data were available for 172 of the cases. The median length of follow-up was 3.6 years (range 0–10). There were five recurrences; three of DCIS and two of invasive breast cancer, giving a recurrence rate of 2.7%. The median time to recurrence was 2 years (range 1–7 years).

The average size of the missing samples was 14.75 mm (range 1–50 mm), which was statistically significantly smaller than that used in the TMAs (*P*=0.04); however, there were no differences in distribution of grades between the missing cores and those used on the TMAs. Of the missing cases, 63.3% of the tumours were HG, 13.3% were IG and 11.7% were LG. Data were missing on the size of the tumours in 32 of the 60 cases and on the grade in 7 of the 60 cases (Table 1).

For the validation series, a further 75 cases of DCIS were analysed. The clinicopathological features of this series showed no significant difference to the test series (data not shown). Follow-up in these patients is limited, therefore, relationship to outcome was not assessed.

### Comparison of TMA samples and whole sections

To evaluate how representative the TMA samples were of the whole-tissue section, the grade of DCIS made on TMA was compared with that on whole sections in 187 cases (according to tissue availability), and ER status was compared in 99 cases.

For grade, the same grade was allocated in 168 cases, giving a concordance rate of 89.8%. Those that differed did so by one grade, with no consistent pattern. For ER status, the score on the whole-tissue section differed from the TMA score in only 10 cases, giving a concordance rate of 94%.

### Expression of biomarkers

**Figure 1:**
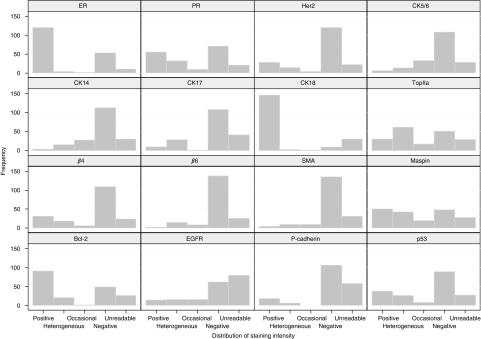
Graphical representation of staining patterns.

The distribution of staining for each marker is shown in Figure 1. Several markers displayed significant heterogeneity within cases, most notably maspin, p53, EGFR and topoisomerase II*α*. However, for the purpose of analysis, any case exhibiting >10% tumour cells staining was designated as positive. Results from at least one of the three DCIS cores were interpretable for between 108 and 178 cases for different markers. Of the interpretable staining, 69.7% cases were positive for ER and 52.1% were positive for PR, and there was a strong correlation between these two markers (Table 2). Her2 was positive in 25.3% of cases and showed an inverse correlation with ER and PR status. Basal CK5/6 and CK14 were expressed in 11.9% and 11.4%, respectively, and were strongly correlated with each other. The highest level of tissue loss was seen with EGFR but data were available on 108 cases, and 27.8% of these showed positive staining.

Using this limited panel of markers, it is possible to allocate samples to the intrinsic molecular subtypes defined by Perou *et al* (2000) and by these surrogate immunohistochemical markers in several studies (Carey *et al*, 2006; Paredes *et al*, 2007). Thus, the DCIS samples were categorised as luminal A (ER+/PR+/Her−), luminal B (ER+PR+ or PR−/Her 2+), Her 2 (ER−/PR−/Her2+) and triple negative (TN; ER−/PR−/Her2−). Within the TNs, we identified a basal-like sub-group (ER−/PR−/Her2−/CK5/6 and/or EGFR+). We found that 38.3% were luminal A, 6.9% were luminal B, 14.9% were Her2 overexpressing and 7.5% were TN. Basal-like tumours comprised 57.1% of TN tumours and 4.2% of the total number of cases. A total of 32.5% was not classifiable owing to lack of information on one or more markers.

A wider analysis of basal and luminal cytoskeletal proteins showed expression of basal CK17 in 25.9% of cases, and the myoepithelial-associated SMA in 8.3% of cases, whereas the luminal CK18 was positive in 93.7% of cases. Other markers previously associated with a basal-like phenotype exhibited varying levels of expression: p53 staining was evident in 40.4% of samples whereas P-cadherin was positive in 20% of cases, and the myoepithelial-associated *β*4-integrin was expressed in 29.9% cases, with *β*6-integrin staining in only 9.8% of samples.

The paired correlations identified between the markers is summarised in Table 2. The basal CKs were inversely correlated with luminal CK and also with ER. A positive association was also demonstrated between *β*4-integrin and basal CK14 and between P-cadherin and p-53 positivity.

Approximately 57.9% of cases that were interpretable showed staining for topoisomerase II*α* and this was inversely correlated with ER and PR, while maspin was expressed in 58.5% cases and showed a moderate association with both basal CK14 and with Her2 positivity (Table 2). The anti-apoptotic Bcl-2 protein was expressed in 69.1% of cases and was strongly positively associated with ER and PR, and negatively associated with basal CKs, Her2, p53, *β*4-integrin and P-cadherin.

Expression of ER, PR, CK14 and Bcl-2 was associated with LG, whereas Her2, p53 and P-cadherin expression was associated with HG. No association was found between other markers and grade.

### Hierarchical cluster analysis

The first cluster analysis, using all 16 markers, did not identify any distinct subgroups. This may, in part, be due to missing data for individual markers on different cases. A second cluster analysis was therefore performed on a reduced set of markers (those that were at least 80% complete and thought to have the most biological relevance), which were ER, PR, Her2, CK5/6, CK14, topoisomerase II*α*, *β*4-integrin, *β*6-integrin, Bcl-2, maspin and p53. This led to the identification of two distinct subgroups.

**Figure 2:**
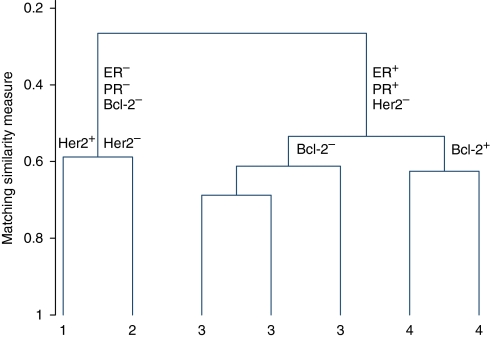
Cluster analysis performed with ER, PR, Her2 and Bcl-2 (*n*=150).

Four markers were the key determinants: ER, PR, Her2 and Bcl-2. Individuals in subgroup 1 were predominantly ER−, PR−, Her2+ and Bcl-2−, and individuals in subgroup 2 were predominantly ER+, PR+, Her2− and Bcl-2+. To maximise the number of individuals included, a third cluster analysis was performed using only the four key markers. This increased the sample size from 128 to 150 and led to four distinct groupings. The associated dendrogram is shown in Figure 2.

Of the 150 cases, 27 (18.0%) fell into group 1 (largely ER−, PR−, Her2+ and Bcl-2−), 19 (12.7%) into group 2 (largely ER−, PR−, Her2− and Bcl-2−), 9 (6%) into group 3 (largely ER+, PR+, Her2− and Bcl-2+) and 7 (4.7%) into group 4 (largely ER+, PR+, Her2− and Bcl-2−). There was a statistically significant association between grade and group (*P*=0.003, Fisher's exact), with group 1 tumours being almost exclusively HG (96%), 84% of group 2 tumours being HG and 63% of group 3 tumours being HG (Table 3). The Cuzick test (Cuzick, 1985) for trend across the groups 1–3 was highly significant (*P*<0.001).

Group 1, therefore, roughly corresponds to the Her2 subtype, group 2 to the TN subtype and both groups 3 and 4 to the luminal A subtype. Within groups 3 and 4, there are a small number of cases that are both ER+ and Her2+ and therefore correspond to the luminal B subtype. In group 1, 10% of tumours were ER positive, in group 2, 42% were ER positive and in group 3, 94% were ER positive. Consequently, it may be that the observed association between group and grade reflects only the known association between ER status and grade. Therefore, to assess the independent prognostic influence of ER, PR, Her2 and Bcl-2 and to investigate whether the relationship between grade and Her2 status is consistent across all levels (i.e., a tumour that is Her2 positive is most likely to be HG, less likely to be of IG and least likely to be of LG), we performed an ordered logistic regression analysis with grade (LG, IG and HG) as the three-level dependent variable and ER, PR, Her2 and Bcl-2 as binary (positive/negative) explanatory variables. Her2 and PR statuses were significant in the multivariate model (*P*=0.03 and *P*=0.02, respectively), suggesting that both Her2 and PR statuses are independent measures of tumour aggressiveness, and that the groups found do provide additional information to ER status alone. Putting all the biomarkers thought to be of biological relevance into the ordered logistic regression led to similar findings (Table 4).

As tumour size is also a measure of disease aggressiveness, we repeated the analysis with size group (1=0–10 mm, 2=11–15 mm, 3=16–30 mm, 4=⩾31 mm) as the dependent variable but there was no significant association with any of the biomarkers.

To examine the robustness of this clustering, a validation set of 75 cases of DCIS was stained for ER, PR, Her2 and Bcl-2, scored in a similar manner and subjected to hierarchical cluster analysis. This identified the same three main groups: ER−, PR−, Her2+ and Bcl-2− (*n*=20); ER−, PR−, Her2− and Bcl-2− (*n*=9); ER+, PR+, Her2− and Bcl-2+ (*n*=43), confirming the robustness of the classification.

## Discussion

Molecular profiling by gene array and its translation into surrogate immunohistochemistry profiles is likely to have a major impact on breast cancer classification and management, and it is important that similar approaches are taken to advance the understanding of DCIS. This study has analysed a large series of pure DCIS samples constructed into TMAs for their expression profile of 16 biomarkers, many of which previously have been shown to distinguish between different subtypes of invasive breast cancer. We demonstrate that each of the intrinsic subtypes identified in invasive breast cancer is also recognised in DCIS, though there are differences in the relative frequencies of the groups in the two stages of disease.

There are practical challenges in using TMAs for the analysis of DCIS, with a higher ‘loss rate’ than is seen with invasive carcinoma, a limitation also reported by others (Tamimi *et al*, 2008). Furthermore, DCIS has been shown to be heterogeneous in nature and grade can vary within a section (Lennington *et al*, 1994; Allred *et al*, 2008). Expression rates of some biological markers have also been shown to vary within whole sections (Allred *et al*, 2008), which indicates that cores used in TMA studies may not be representative of the whole tumour. To overcome this sampling bias, three cores of tissue were analysed from each tumour, an approach validated by other groups studying invasive breast cancers (Zhang *et al*, 2003; Selvarajan *et al*, 2006; Yang *et al*, 2006; Drev *et al*, 2008). A comparison of DCIS grade and ER status was made between TMAs and whole-tumour sections, and this showed ∼90% concordance between the two sample types for both parameters, suggesting that three cores are reasonably representative of whole sections.

The same scoring system was used for all markers to allow direct comparison between markers. Samples were either scored as positive when >70% of cells expressed the marker, heterogeneous when 10–70% of cells expressed the marker, occasional when a few individual cells expressed the marker or negative when no staining was seen. In invasive breast cancers, the Quick score (Leake *et al*, 2000) is used to score ER expression, and variable systems have been used to score other biomarkers, including a cutoff of >10% as positive (Rakha *et al*, 2007). There is currently no consensus on the scoring of biomarkers in DCIS, though a similar percentage cutoff for ER is used in some clinical trials (Cuzick, 2008). Thus, the scoring system is in keeping with other published studies, allowing some level of comparison.

According to the criteria of Carey *et al* (2006), this study showed a frequency of 38.3% for luminal A, 6.9% luminal B, 14.9% Her2, 7.5% TN and 4.2% basal-like in this series of DCIS cases. In invasive breast cancer, frequencies between 58–75% for luminal A, 11–16% for luminal B, 3–6% for Her2 and 11–20% for TN/basal have been reported (Carey *et al*, 2006; Kurebayashi *et al*, 2007; Rakha *et al*, 2007; Kwan *et al*, 2009). Thus, in keeping with previous reports (Park *et al*, 2006), we show a higher frequency of Her2-positive DCIS compared with invasive disease, but lower frequency of TN and basal subtypes.

Meijnen *et al* (2008) carried out an immunohistochemical study on 163 cases of pure DCIS analysing 16 markers. Interestingly, they showed no evidence of CK14 or EGFR expression in their series of DCIS, with CK5/6 being expressed in only three cases.

In an unsupervised cluster analysis, they identified two major groups, an ER+/Bcl-2+ group designated as ‘luminal’, and an ER−/Bcl-2− group designated as non-luminal, in keeping with the results of this study. The luminal subgroup was further subdivided into AR+, AR− and mixed groups, whereas the non-luminal group was subdivided into Her2+ and Her2− subsets. There was a significant relationship between grade and distribution between subsets, with significantly more well-grade and IG DCIS clustering in the luminal groups compared with non-luminal groups. They demonstrated that the IG DCIS shared more features with well-differed than poorly differentiated DCIS and show that the Bcl-2-positive luminal subgroup may indicate a more favourable group of lesions. This is in keeping with the unsupervised hierarchical cluster analysis in the current study, which also separated DCIS into ER−, PR−, Her2−, Bcl-2− and ER+, PR+, Her2−, Bcl-2+ subtypes, with a further ER−, Her2− group. Furthermore, Meijnen *et al* also report a low frequency of basal-like/TN DCIS cases, however they define that phenotype. Thus, CK14 was not detected in any of their cases, and CK5/6 in only three cases. When using the TN definition, only eight cases were found to be ER/PR/Her2 negative.

This is in contrast to a study by Livasy *et al* (2007), who, in an analysis of 245 pure DCIS cases, identified 8% as basal (defined as ER−, Her2−, EGFR+ and/or CK5/6+) and a further 6% fell into the TN category. This frequency of 14% correlates with the frequency of the basal/TN category in sporadic invasive breast cancers. These differences may be related to analysis of whole-tissue sections *vs* TMAs and reflect the heterogeneity reported in DCIS, or may reflect a different spectrum of DCIS analysed. Tamimi *et al* (2008) also report significant differences between molecular phenotypes in DCIS and invasive breast cancer. In a large study comparing the immunoprofile of invasive breast cancer (*n*=2249) and their associated DCIS (*n*=272), they showed that the basal subtype (defined as ER/PR/Her2 negative, positive for EGFR and for CK5/6) was less frequent in DCIS compared with invasive cancers (7.7 of 10.9%), and more so when restricted to invasive ductal carcinoma. Furthermore, the Her2+ subtype was significantly more frequent in DCIS compared with invasive disease (13.6 of 5.5%), as previously reported, as was the luminal B subtype (defined as ER+/Her2+), reported as 13.2% frequency in DCIS compared with 5.2% in invasive cancers.

As a result of the application of different definitions for subtypes, in particular the basal/TN category, it is difficult to make direct comparisons between studies; however, Tamimi *et al* (2008) and Livasy *et al* (2007) use the same definition for basal, and report frequencies in DCIS of 7.7 and 8%, in contrast to the very low frequency (∼1.8%) reported on the basis of CK5/6 staining by Meijnen *et al* (2008). Hannemann *et al* (2006) also found the basal phenotype to be infrequent in DCIS, with only 2 of 39 cases showing expression of CK5/6.

Owing to the relatively low rate of recurrence of DCIS, either as *in situ* or invasive disease, there are a few studies that relate molecular phenotype or biomarker expression of DCIS to the outcome. In most studies, results are extrapolated from knowledge of associations with behaviour in invasive breast cancer. Thus, it is well recognised that the basal/TN subtype is associated with more aggressive disease in invasive cancers, therefore, it is postulated that DCIS with a basal phenotype is likely to exhibit more aggressive behaviour. This is not a foregone conclusion and other factors may be at play, such as microenvironmental changes, that may be of more importance in DCIS than established invasive disease.

Few studies have undertaken genomic analysis of DCIS. One of the largest studies to date is reported by Hannemann *et al* (2006), who carried out gene expression microarray analysis of 39 cases of DCIS. An unsupervised cluster analysis identified two major subgroups, a predominantly ER+ group and a predominantly ER– group. Discrimination of a Her2+ subgroup was not as clear, and they could not identify a significant basal-type group, which would appear to support the IHC studies, suggesting that this phenotype is infrequent in DCIS. In an analysis to identify a gene classifier that could distinguish between LG and HG DCIS, Bcl-2 was one of the top genes being upregulated in the well-differentiated samples, again, correlating with the results reported in the current study. It is also of note that Bcl-2 has recently been reported as a powerful independent predictor of good prognosis in a study of >11 000 invasive breast carcinomas (Dawson *et al*, 2010), and this further suggests that the clustering of DCIS into subgroups defined in part by Bcl-2 may be of functional and clinical importance.

## Conclusions

The results of this study indicate that it may be possible to classify DCIS according to its immunohistochemical expression profile, and that individual markers act independently of ER in associating with grade. The relative frequency of molecular subtypes in DCIS *vs* invasive disease provides some insight into the impact of molecular subtype on progression. This is important as, if a group of patients with very low-risk DCIS could be identified, the over-treatment of women, particularly in the breast-screening setting, could be reduced, and the associated psychological and physical harms thereby minimised or avoided.

## Figures and Tables

**Table 1:** Patient, tumour and treatment characteristics

	**TMA cases (*N*=188)**		**Missing cases (*N*=60)**	
**Characteristic**	**Number**	**%**	**Number**	**%**
Mean age, years (range)	56 (25–85)		66 (43–95)	
				
*Laterality*				
Left breast	95	50.5	22	44
Right breast	89	47.4	28	56
Unknown	4	2.1	9	18
				
*Primary surgical treatment*				
Mastectomy alone	14	7.4	3	5.1
+Axillary node clearance	8	4.3	0	0
+Sentinel node procedure	42	22.3	1	1.7
Breast-conserving surgery	100	53.2	29	49.2
+Axillary node clearance	0	0	1	1.7
+Sentinel node procedure	12	6.4	12	20.3
Excision biopsy	9	4.8	3	5.1
Unknown	3	1.6	11	16.9
				
*Secondary surgical treatment*				
Mastectomy	35	18.6	10	16.9
Breast-conserving surgery	25	13.2	11	18.6
				
*Third surgical treatment*				
Mastectomy	8	4	2	3.4
Breast-conserving surgery	2	1	0	0
				
*Lesion size*				
1–10 mm	19	10.1	11	18.3
11–20 mm	35	18.6	7	11.7
21–30 mm	17	9.0	2	3.3
31–40 mm	11	5.9	0	0
41–50 mm	8	4.3	2	3.3
>50 mm	7	4.3	0	0
Multicentric/multifocal	17	9.0	5	8.3
Extensive	10	5.3	1	1.8
Not stated	63	33.5	32	53.3
				
*Grade*				
Low	20	10.6	7	11.7
Intermediate	25	13.3	8	13.3
High	140	74.5	38	63.3
Unknown	3	1.6	7	11.7
				
*Adjuvant therapy*				
Radiotherapy				
Yes	22	11.7	8	13.3
No	145	77.1	43	71.7
Unknown	21	11.2	9	15.0
Endocrine				
Yes	80	42.6	22	36.7
No	88	46.8	29	48.3
Unknown	20	10.6	9	15.0

Abbreviation: TMA=tissue microarray.

**Table 2:** Pairwise tetrachoric correlation coefficients indicating the strength and direction of associations between staining positive for one biomarker and staining positive for another

	**ER**	**PR**	**Her2**	**CK56**	**CK14**	**CK17**	**CK18**	**TopIIa**	***β*4**	***β*6**	**SMA**	**Maspin**	**Bcl-2**	**EGFR**	**P-cad-herin**	**p53**
ER	1.00															
PR	0.81	1.00														
Her2	−0.71	−0.62	1.00													
CK56	−0.03	0.36	0.00	1.00												
CK14	−0.42	−0.26	0.14	0.81	1.00											
CK17	−0.02	0.02	0.04	0.13	0.30	1.00										
CK18	0.42	0.02	1.00	1.00	−0.21	−0.06	1.00									
TopIIa	−0.11	−0.41	0.29	−0.17	0.09	−0.18	−0.09	1.00								
*β*4	−0.00	0.01	0.34	0.53	0.61	0.07	0.09	0.29	1.00							
*β*6	−0.52	−0.60	0.47	−0.15	−0.04	0.23	1.00	0.11	−0.11	1.00						
SMA	0.03	−0.22	0.12	0.71	0.78	0.05	1.00	−1.00	−0.07	−1.00	1.00					
Maspin	−0.04	−0.16	0.45	0.05	0.38	0.32	0.36	0.19	−0.19	0.45	1.00	1.00				
Bcl-2	0.86	0.60	−0.67	0.14	−0.01	−0.09	0.68	−0.14	−0.09	−0.45	−0.01	0.05	1.00			
EGFR	−0.08	−0.34	−0.30	−1.00	−0.13	0.08	−0.17	0.39	0.02	0.04	−1.00	0.01	−0.28	1.00		
P-cadherin	−0.78	−0.77	0.66	−0.18	0.47	0.18	−0.47	0.47	0.27	0.11	0.19	0.04	−0.73	−0.20	1.00	
p53	−0.34	−0.47	0.50	−0.07	−0.13	0.33	0.13	0.37	0.05	0.42	−1.00	0.10	−0.44	0.09	0.21	1.00

*Notes*:

(1) A positive value for correlation indicates that being positive for one marker is associated with being positive for the other, and vice versa.

(2) A negative value for correlation indicates that being positive for one marker is associated with being negative for the other, and vice versa.

(3) An absolute value for the correlation coefficient of between 0.1 and 0.3 indicates weak correlation, between 0.3 and 0.5 indicates moderate correlation and between 0.5 and 1 indicates strong correlation.

**Table 3:** Comparison of percentage positive cases for each marker with those available in the literature

	**Published literature**			
**Marker**	**Normal breast**	**DCIS** **% positive**	**Source**	**% of cases expressing marker in this study**
ER	Low levels of expression	57–88	(Malafa *et al*, 1990; Chaudhuri *et al*, 1993; Suo *et al*, 2001)	60.2
PR	Low levels of expression	40–80	(Suo *et al*, 2001; Bryan *et al*, 2006)	46.4
Her2	Not expressed	34–67	(Suo *et al*, 2001; Bryan *et al*, 2006)	22.4
EGFR	Myoepithelial cells	8.2–94	(Suo *et al*, 2001; Bryan *et al*, 2006; Dabbs *et al*, 2006)	17.3
CK5/6	Myoepithelial cells	3.7–24	(Otterbach *et al*, 2000; Abd El-Rehim *et al*, 2004a; Bryan *et al*, 2006)	10.2
CK14	Myoepithelial cells	19.4–24	(Abd El-Rehim *et al*, 2004a; Bryan *et al*, 2006)	9.2
CK17	Myoepithelial cells	32	(Abd El-Rehim *et al*, 2004a; Bryan *et al*, 2006)	19.4
CK18	Luminal cells	88	(Abd El-Rehim *et al*, 2004a)	79.1
SMA	Myoepithelial cells	43.8	(Rakha *et al*, 2006)	6.6
p53	Mutation not expressed	10–20	(Done *et al*, 2001; Ribeiro-Silva *et al*, 2003; Malamou-Mitsi *et al*, 2006)	35.2
TopIIa	Low levels of expression	6.8	(Shpitz *et al*, 2000; Fritz *et al*, 2005)	49.0
Bcl-2	Myoepithelial cells	52–81.8	(Malamou-Mitsi *et al*, 2006; Park *et al*, 2006; Rolland *et al*, 2007)	47.5
Maspin	Myoepithelial cells	9.6	(Zou *et al*, 1994; Umekita *et al*, 2002)	50.7
*β*4	Myoepithelial cells	18	(Hanby *et al*, 1993)	19.9
*β*6	Not expressed	—	(Arihiro *et al*, 2000)	9.2
P-Cad	Myoepithelial cells	25–100	(Peralta Soler *et al*, 1999; Kovacs *et al*, 2003; Paredes *et al*, 2007)	12.8

Abbreviation: DCIS=ductal carcinoma *in situ*.

**Table 4:** Odds ratios for grade (low, intermediate, high) from the multivariate ordered logistic regression analysis (*n*=148)

**Biomarker**	**No. (negative/positive)**	**Odds ratio (univariate)**	**Odds ratio (multivariate)**	**95% Confidence interval**	***P*-value**
ER	44/104	0.17	0.88	0.22–3.57	0.86
PR	68/80	0.21	0.34	0.12–0.92	0.03
Bcl-2	47/101	0.22	0.61	0.17–2.22	0.45
Her2	109/39	10.38	12.45	1.55–99.98	0.02

## References

[bib1] Abd El-Rehim DM, Pinder SE, Paish CE, Bell JA, Blamey RW, Robertson JF, Nicholson RI, Ellis IO (2004a) Expression of luminal and basal cytokeratins in human breast carcinoma. J Pathol 203(2): 661–671 15141381 10.1002/path.1559

[bib2] Abd El-Rehim DM, Pinder SE, Paish CE, Bell JA, Rampaul RS, Blamey RW, Robertson JF, Nicholson RI, Ellis IO (2004b) Expression and co-expression of the members of the epidermal growth factor receptor (EGFR) family in invasive breast carcinoma. Br J Cancer 91: 1532–1542 15480434 10.1038/sj.bjc.6602184 PMC2410019

[bib3] Allred DC, Wu Y, Mao S, Nagtegaal ID, Lee S, Perou CM, Mohsin SK, O’Connell P, Tsimelzon A, Medina D (2008) Ductal carcinoma *in situ* and the emergence of diversity during breast cancer evolution. Clin Cancer Res 14: 370–378 18223211 10.1158/1078-0432.CCR-07-1127

[bib4] Arihiro K, Kaneko M, Fujii S, Inai K, Yokosaki Y (2000) Significance of alpha 9 beta 1 and alpha v beta 6 integrin expression in breast carcinoma. Breast Cancer 7: 19–26 11029766 10.1007/BF02967183

[bib5] Bryan BB, Schnitt SJ, Collins LC (2006) Ductal carcinoma *in situ* with basal-like phenotype: a possible precursor to invasive basal-like breast cancer. Mod Pathol 19: 617–621 16528377 10.1038/modpathol.3800570

[bib6] Calinski T (1973) A dendrite method for cluster analysis. Commun Stat 3: 1–27

[bib7] Carey LA, Perou CM, Livasy CA, Dressler LG, Cowan D, Conway K, Karaca G, Troester MA, Tse CK, Edmiston S, Deming SL, Geradts J, Cheang MC, Nielsen TO, Moorman PG, Earp HS, Millikan RC (2006) Race, breast cancer subtypes, and survival in the Carolina Breast Cancer Study. JAMA 295: 2492–2502 16757721 10.1001/jama.295.21.2492

[bib8] Chaudhuri B, Crist KA, Mucci S, Malafa M, Chaudhuri PK (1993) Distribution of estrogen receptor in ductal carcinoma *in situ* of the breast. Surgery 113: 134–137 8381562

[bib9] Cheang MC, Voduc D, Bajdik C, Leung S, McKinney S, Chia SK, Perou CM, Nielsen TO (2008) Basal-like breast cancer defined by five biomarkers has superior prognostic value than triple-negative phenotype. Clin Cancer Res 14: 1368–1376 18316557 10.1158/1078-0432.CCR-07-1658

[bib10] Cuzick J (1985) A Wilcoxon-type test for trend. Stat Med 4: 87–90 3992076 10.1002/sim.4780040112

[bib11] Cuzick J (2008) IBIS II: a breast cancer prevention trial in postmenopausal women using the aromatase inhibitor anastrazole. Expert Rev Anticancer Ther 8: 1377–1385 18759690 10.1586/14737140.8.9.1377

[bib12] Dabbs DJ, Chivukula M, Carter G, Bhargava R (2006) Basal phenotype of ductal carcinoma *in situ*: recognition and immunohistologic profile. Mod Pathol 19: 1506–1511 16941011 10.1038/modpathol.3800678

[bib13] Dawson SJ, Makretsov N, Blows FM, Driver KE, Provenzano E, Le Quesne J, Baglietto L, Severi G, Giles GG, McLean CA, Callagy G, Green AR, Ellis I, Gelmon K, Turashvili G, Leung S, Aparicio S, Huntsman D, Caldas C, Pharoah P (2010) BCL2 in breast cancer: a favourable prognostic marker across molecular subtypes and independent of adjuvant therapy received. Br J Cancer 103(5): 668–675 20664598 10.1038/sj.bjc.6605736 PMC2938244

[bib14] Done SJ, Arneson CR, Ozcelik H, Redston M, Andrulis IL (2001) P53 protein accumulation in non-invasive lesions surrounding p53 mutation positive invasive breast cancers. Breast Cancer Res Treat 65: 111–118 11261826 10.1023/a:1006425809069

[bib15] Drev P, Grazio SF, Bracko M (2008) Tissue microarrays for routine diagnostic assessment of HER2 status in breast carcinoma. Appl Immunohistochem Mol Morphol 16: 179–184 18227723 10.1097/PAI.0b013e31806875e1

[bib16] Duda RO (1973) Pattern Classification and Scene Analysis. Wiley: New York

[bib17] Fritz P, Cabrera CM, Dippon J, Gerteis A, Simon W, Aulitzky WE, van der Kuip H (2005) c-erbB2 and topoisomerase IIalpha protein expression independently predict poor survival in primary human breast cancer: a retrospective study. Breast Cancer Res 7: R374–R384 15987433 10.1186/bcr1012 PMC1143560

[bib18] Hanby AM, Gillett CE, Pignatelli M, Stamp GW (1993) Beta 1 and beta 4 integrin expression in methacarn and formalin-fixed material from *in situ* ductal carcinoma of the breast. J Pathol 171: 257–262 7512643 10.1002/path.1711710405

[bib19] Hannemann J, Velds A, Halfwerk JB, Kreike B, Peterse JL, van de Vijver MJ (2006) Classification of ductal carcinoma *in situ* by gene expression profiling. Breast Cancer Res 8: R61 17069663 10.1186/bcr1613 PMC1779498

[bib20] Jones JL (2006) Overdiagnosis and overtreatment of breast cancer: progression of ductal carcinoma *in situ*: the pathological perspective. Breast Cancer Res 8: 204 16677423 10.1186/bcr1397 PMC1557717

[bib21] Kovacs A, Dhillon J, Walker RA (2003) Expression of P-cadherin, but not E-cadherin or N-cadherin, relates to pathological and functional differentiation of breast carcinomas. Mol Pathol 56: 318–322 14645693 10.1136/mp.56.6.318 PMC1187349

[bib22] Kurebayashi J, Moriya T, Ishida T, Hirakawa H, Kurosumi M, Akiyama F, Kinoshita T, Takei H, Takahashi K, Ikeda M, Nakashima K (2007) The prevalence of intrinsic subtypes and prognosis in breast cancer patients of different races. Breast 16(Suppl 2): S72–S77 17714947 10.1016/j.breast.2007.07.017

[bib23] Kwan ML, Kushi LH, Weltzien E, Maring B, Kutner SE, Fulton RS, Lee MM, Ambrosone CB, Caan BJ (2009) Epidemiology of breast cancer subtypes in two prospective cohort studies of breast cancer survivors. Breast Cancer Res 11: R31 19463150 10.1186/bcr2261 PMC2716499

[bib24] Leake R, Barnes D, Pinder S, Ellis I, Anderson L, Anderson T, Adamson R, Rhodes T, Miller K, Walker R (2000) Immunohistochemical detection of steroid receptors in breast cancer: a working protocol. UK Receptor Group, UK NEQAS, The Scottish Breast Cancer Pathology Group, and The Receptor and Biomarker Study Group of the EORTC. J Clin Pathol 53: 634–635 11002770 10.1136/jcp.53.8.634 PMC1762930

[bib25] Lennington WJ, Jensen RA, Dalton LW, Page DL (1994) Ductal carcinoma *in situ* of the breast. Heterogeneity of individual lesions. Cancer 73: 118–124 8275415 10.1002/1097-0142(19940101)73:1<118::aid-cncr2820730121>3.0.co;2-r

[bib26] Livasy CA, Perou CM, Karaca G, Cowan DW, Maia D, Jackson S, Tse CK, Nyante S, Millikan RC (2007) Identification of a basal-like subtype of breast ductal carcinoma *in situ*. Hum Pathol 38: 197–204 17234468 10.1016/j.humpath.2006.08.017

[bib27] Lu S, Simin K, Khan A, Mercurio AM (2008) Analysis of integrin beta4 expression in human breast cancer: association with basal-like tumors and prognostic significance. Clin Cancer Res 14: 1050–1058 18281537 10.1158/1078-0432.CCR-07-4116

[bib28] Makretsov NA, Huntsman DG, Nielsen TO, Yorida E, Peacock M, Cheang MC, Dunn SE, Hayes M, van de Rijn M, Bajdik C, Gilks CB (2004) Hierarchical clustering analysis of tissue microarray immunostaining data identifies prognostically significant groups of breast carcinoma. Clin Cancer Res 10: 6143–6151 15448001 10.1158/1078-0432.CCR-04-0429

[bib29] Malafa M, Chaudhuri B, Thomford NR, Chaudhuri PK (1990) Estrogen receptors in ductal carcinoma *in situ* of breast. Am Surg 56: 436–439 2164337

[bib30] Malamou-Mitsi V, Gogas H, Dafni U, Bourli A, Fillipidis T, Sotiropoulou M, Vlachodimitropoulos D, Papadopoulos S, Tzaida O, Kafiri G, Kyriakou V, Markaki S, Papaspyrou I, Karagianni E, Pavlakis K, Toliou T, Scopa C, Papakostas P, Bafaloukos D, Christodoulou C, Fountzilas G (2006) Evaluation of the prognostic and predictive value of p53 and Bcl-2 in breast cancer patients participating in a randomized study with dose-dense sequential adjuvant chemotherapy. Ann Oncol 17: 1504–1511 16968874 10.1093/annonc/mdl147

[bib31] Matos I, Dufloth R, Alvarenga M, Zeferino LC, Schmitt F (2005) p63, cytokeratin 5, and P-cadherin: three molecular markers to distinguish basal phenotype in breast carcinomas. Virchows Arch 447: 688–694 16012853 10.1007/s00428-005-0010-7

[bib32] Meijnen P, Peterse JL, Antonini N, Rutgers EJ, van de Vijver MJ (2008) Immunohistochemical categorisation of ductal carcinoma *in situ* of the breast. Br J Cancer 98: 137–142 18043578 10.1038/sj.bjc.6604112 PMC2359678

[bib33] Nielsen TO, Hsu FD, Jensen K, Cheang M, Karaca G, Hu Z, Hernandez-Boussard T, Livasy C, Cowan D, Dressler L, Akslen LA, Ragaz J, Gown AM, Gilks CB, van de Rijn M, Perou CM (2004) Immunohistochemical and clinical characterization of the basal-like subtype of invasive breast carcinoma. Clin Cancer Res 10: 5367–5374 15328174 10.1158/1078-0432.CCR-04-0220

[bib34] Otterbach F, Bankfalvi A, Bergner S, Decker T, Krech R, Boecker W (2000) Cytokeratin 5/6 immunohistochemistry assists the differential diagnosis of atypical proliferations of the breast. Histopathology 37: 232–240 10971699 10.1046/j.1365-2559.2000.00882.x

[bib35] Paredes J, Lopes N, Milanezi F, Schmitt FC (2007) P-cadherin and cytokeratin 5: useful adjunct markers to distinguish basal-like ductal carcinomas *in situ*. Virchows Arch 450: 73–80 17123107 10.1007/s00428-006-0334-y

[bib36] Park K, Han S, Kim HJ, Kim J, Shin E (2006) HER2 status in pure ductal carcinoma *in situ* and in the intraductal and invasive components of invasive ductal carcinoma determined by fluorescence *in situ* hybridization and immunohistochemistry. Histopathology 48: 702–707 16681686 10.1111/j.1365-2559.2006.02403.x

[bib37] Peralta Soler A, Knudsen KA, Salazar H, Han AC, Keshgegian AA (1999) P-cadherin expression in breast carcinoma indicates poor survival. Cancer 86: 1263–1272 10506713 10.1002/(sici)1097-0142(19991001)86:7<1263::aid-cncr23>3.3.co;2-u

[bib38] Perou CM, Sorlie T, Eisen MB, van de Rijn M, Jeffrey SS, Rees CA, Pollack JR, Ross DT, Johnsen H, Akslen LA, Fluge O, Pergamenschikov A, Williams C, Zhu SX, Lonning PE, Borresen-Dale AL, Brown PO, Botstein D (2000) Molecular portraits of human breast tumours. Nature 406: 747–752 10963602 10.1038/35021093

[bib39] Rakha EA, El-Sayed ME, Green AR, Lee AH, Robertson JF, Ellis IO (2007) Prognostic markers in triple-negative breast cancer. Cancer 109: 25–32 17146782 10.1002/cncr.22381

[bib40] Rakha EA, Putti TC, Abd El-Rehim DM, Paish C, Green AR, Powe DG, Lee AH, Robertson JF, Ellis IO (2006) Morphological and immunophenotypic analysis of breast carcinomas with basal and myoepithelial differentiation. J Pathol 208: 495–506 16429394 10.1002/path.1916

[bib41] Ribeiro-Silva A, Zambelli Ramalho LN, Britto Garcia S, Zucoloto S (2003) The relationship between p63 and p53 expression in normal and neoplastic breast tissue. Arch Pathol Lab Med 127: 336–340 12653579 10.5858/2003-127-0336-TRBPAP

[bib42] Rolland P, Spendlove I, Madjd Z, Rakha EA, Patel P, Ellis IO, Durrant L (2007) The p53 positive Bcl-2 negative phenotype is an independent marker of prognosis in breast cancer. Int J Cancer 120: 1311–1317 17187363 10.1002/ijc.22430

[bib43] Selvarajan S, Tan SY, Sii LH, Tan PH (2006) c-erbB-2 (HER-2/neu) immunohistochemistry in invasive breast cancer: is there concordance between standard sections and tissue microarrays? Pathology 38: 316–320 16916720 10.1080/00313020600820872

[bib44] Shpitz B, Bomstein Y, Zehavi T, Bernheim J, Liverant S, Kaufman Z, Buklan G, Klein E (2000) Topoisomerase IIalpha expression in ductal carcinoma *in situ* of the breast: a preliminary study. Hum Pathol 31: 1249–1254 11070118 10.1053/hupa.2000.19297

[bib45] Sorlie T, Perou CM, Tibshirani R, Aas T, Geisler S, Johnsen H, Hastie T, Eisen MB, van de Rijn M, Jeffrey SS, Thorsen T, Quist H, Matese JC, Brown PO, Botstein D, Eystein Lonning P, Borresen-Dale AL (2001) Gene expression patterns of breast carcinomas distinguish tumor subclasses with clinical implications. Proc Natl Acad Sci USA 98: 10869–10874 11553815 10.1073/pnas.191367098 PMC58566

[bib46] Suo Z, Berner HS, Risberg B, Karlsson MG, Nesland JM (2001) Estrogen receptor-alpha and C-ERBB-4 expression in breast carcinomas. Virchows Arch 439: 62–69 11499842 10.1007/s004280000392

[bib47] Tamimi RM, Baer HJ, Marotti J, Galan M, Galaburda L, Fu Y, Deitz AC, Connolly JL, Schnitt SJ, Colditz GA, Collins LC (2008) Comparison of molecular phenotypes of ductal carcinoma *in situ* and invasive breast cancer. Breast Cancer Res 10: R67 18681955 10.1186/bcr2128 PMC2575540

[bib48] Umekita Y, Ohi Y, Sagara Y, Yoshida H (2002) Expression of maspin predicts poor prognosis in breast-cancer patients. Int J Cancer 100: 452–455 12115529 10.1002/ijc.10500

[bib49] Yang XR, Charette LA, Garcia-Closas M, Lissowska J, Paal E, Sidawy M, Hewitt SM, Rimm DL, Sherman ME (2006) Construction and validation of tissue microarrays of ductal carcinoma *in situ* and terminal duct lobular units associated with invasive breast carcinoma. Diagn Mol Pathol 15: 157–161 16932071 10.1097/01.pdm.0000213453.45398.e0

[bib50] Zhang D, Salto-Tellez M, Putti TC, Do E, Koay ES (2003) Reliability of tissue microarrays in detecting protein expression and gene amplification in breast cancer. Mod Pathol 16: 79–84 12527717 10.1097/01.MP.0000047307.96344.93

[bib51] Zou Z, Anisowicz A, Hendrix MJ, Thor A, Neveu M, Sheng S, Rafidi K, Seftor E, Sager R (1994) Maspin, a serpin with tumor-suppressing activity in human mammary epithelial cells. Science 263: 526–529 8290962 10.1126/science.8290962

