# Supplementary material for: Molecular subtyping of DCIS: heterogeneity of breast cancer reflected in pre-invasive disease
Source: Br J Cancer. 2010 Dec 7;104(1):120–7. doi: 10.1038/sj.bjc.6606021 (PMC3039794; doi:10.1038/sj.bjc.6606021)
Supplement: Supplementary Table 1 [file 6606021x1.doc]

Supplementary Table 1:

Antibodies and Antigen retrieval techniques employed in immunohistochemistry.

| **Antibody** | **Manufacturer** | **Clone** | **Dilution** | **Species** | **Antigen retrieval** |
| --- | --- | --- | --- | --- | --- |
| ER | Novocastra | NCL-L-ER-6F11 | 1:40 | Mouse | Citrate Buffer |
| PR | Novocastra | 16 | 1:100 | Mouse | Citrate Buffer |
| Her2 | Novocastra | 10A7 | 1:50 | Mouse | None |
| EGFR | Dako | K1492 | Pre-mixed | Mouse | Proteinase K |
| CK 5/6 | Dakocytomation | D5/16B4 | 1:50 | Mouse | Citrate Buffer |
| CK 14 | Serotec | LL002 | 1:5 | Mouse | Citrate Buffer |
| CK 17 | Sigma | CK-E3 | 1:50 | Mouse | Proteinase K |
| CK 18 | Serotec | CY90 | Neat | Mouse | Citrate Buffer |
| SMA | Dakocytomation | 1A4 | 1:50 | Mouse | Citrate Buffer |
| p53 | Novocastra | NCL-p53-DO7 | 1:50 | Mouse | Citrate Buffer |
| Topoisomerase IIα | Novocastra | 3F6 | 1:40 | Mouse | Citrate Buffer |
| Bcl-2 | Abcam | 100/D5 | 1:50 | Mouse | Citrate Buffer |
| Maspin | Pharmingen | G167-70 | 1:500 | Mouse | Citrate Buffer |
| β4 integrin | Chemicon | 439-9B | 1:400 | Rat | Proteinase K |
| β6 integrin | Gift from J Marshall | 62G2 | 1:4200 | Rat | Pepsin |
| P-cadherin | BD Transduction labs | 56 | 1:50 | Mouse | Vector antigen unmasking solution |
